# 

**Published:** 1915-11-20

**Authors:** 


### St. Thomas's Hospital.

The following house appointments have been made: *Casualty Officers and Resident Anæsthetists.*—Messrs. W. H. C. Romains, B.A. Cantab., M.R.C.S., L.R.C.P.; G. T. Gimlette, B.A. Oxon., M.R.C.S., L.R.C.P.; G. M. Vevers, M.R.C.S., L.R.C.P.; C. Gardiner Hill, B.A. Cantab., M.R.C.S., L.R.C.P.; W. Thomas, B.Sc. Lond., M.R.C.S., L.R.C.P.; G. C. Berg, M.R.C.S., L.R.C.P.; C. H. C. Byrne, M.R.C.S., L.R.C.P. *Obstetric House Physicians.*—(Senior) Mr. W. H. Marshall, B.A. Cantab., M.R.C.S., L.R.C.P.; (Junior) Mr. F. E. Higgins, B.A. Cantab., M.R.C.S., L.R.C.P. *Clinical Assistant, Children's Medical Department.*—Mr. S. L. Bhatia, B.A. Cantab., M.R.C.S., L.R.C.P.

### Buildings Contemplated.

Aldershot.—The Urban District Council have been informed by the Local Government Board that they will in due course issue their formal sanction to a loan for the proposed extensions at the isolation hospital, and that they will raise no objection to the work being started at once.

Chesterton.—The extensions to the Bradwell Joint Isolation Hospital at Chesterton have been formally opened. The scheme was carried out under the supervision of Messrs. A. R. Wood and Son, architects. The cost is estimated at £7,500.

### Personalia.

Dr. Varrier Jones has been appointed whole-time tuberculosis officer for Cambridgeshire, at a salary of £500 a year.

Mr. H. Underdown has given his country house, Buckenham Hall, Norfolk, to the British Red Cross Society for hospital purposes.

The Local Government Board decline to consent to Dr. Fraser, medical officer of health for Chesterfield, joining the R.A.M.C. unless he provides an efficient substitute.

Mr. H. O. Williams, M.R.C.S., L.R.C.P., has been appointed medical officer of the Milford district of the Haverfordwest Guardians. Mr. Williams was formerly medical officer for Pembrokeshire.

### PASSING EVENTS AND TOPICS.

### No Red Cross.

From the front cover of the current number of the *South African Nursing Record* the familiar red cross has disappeared. Its removal is enforced and not voluntary, the use of the red cross for private purposes, without special permission, being now illegal.

### Alexandra Day in Leicester.

The income and expenditure account, duly audited, has been issued by the local Alexandra Day Control Committee, and shows total receipts £1,934 19s. 3d., total expenses £701 9s. 7d. The balance, £1,233 9s. 8d., has been divided among the following local charities: The Surgical Aid Society, £50; the Children's Hospital in connection with the Leicester Royal Infirmary, 40 per cent., £473 7s. 10d.; the Cripples' Guild, 15 per cent., £177 10s. 6d.; the Mission to the Deaf and Dumb, 25 per cent., £295 17s. 5d.; the Maternity Hospital, 20 per cent., £236 13s. 11d.

### A County Council Medical Committee.

The L.C.C. announces the appointment of a Joint Sub-Committee, comprising five members each of the Establishment and Education Committees and two members of the Public Health Committee, to deal with the selection for appointment and to advise on questions relating to the conduct, pay, promotion, etc., of the medical assistants engaged primarily for education work, for school nurses in the public health department, and the organising staff of the children's care work in the public health and education departments. The members of the Joint Sub-Committee are Dr. Sophia Jevons, Mrs. Wilton Phipps, Miss S. Lawrence, and Messrs. E. R. Debenham, H. J. Greenwood, I. Salmon, E. Smith, R. L. Stuart, F. R. Anderton, D. Blackley, Cyril Cobb, and O. E. Warburg.

### The German Doctors' Ten Commandments.

Consequent upon the depletion of the ranks of German doctors owing to the demands of the war, civilian practitioners who are left at home are naturally overworked. In order that the pressure may be relieved a little, the Medical Society of Frankfort has drawn up ten rules to govern the relations between the doctors and the public. These rules have been distributed among patients, and are also posted up in the practitioners' waiting-rooms. The object is to impress upon the public the necessity for sparing the medical man as much as possible. Thus patients are commanded not to call the doctor unnecessarily. Another rule is "Never call the doctor at night except in an emergency case," and another, "On Sunday the doctor should be allowed to rest." On the whole the ten commandments are based on common-sense principles.

### French School Buildings as Hospitals.

The French authorities have come to the conclusion that there are sound reasons against the retention of school buildings as military hospitals, and an arrangement has been made by which a certain number of schools utilised up to the present for the treatment of wounded will be reopened for their original purpose. Other buildings will be substituted for the restored schools, and in no district will the number of beds be reduced. The desire of the authorities is to reconcile the welfare of the wounded with the interests of education.

### Medals for Nurses.

At the monthly meeting of the Board of Management of King Edward VII.'s Hospital, Cardiff, Colonel Bruce Vaughan reported that Sir William James Thomas had most generously offered a sum of £200 to be used as an endowment fund, the interest to be applied to the purchase each year of a gold and silver medal to be awarded to the best nurses of each year. General Lee, in proposing a vote of thanks to Sir William, likened him to one of the good fairies one reads about who went about trying to discover what people wanted in order to give it to them. Colonel Bruce Vaughan and Dr. Herbert Vachell, the chairman of the nursing committee, regarded the provision of such medals as fulfilling a useful purpose.

### Gifts and Bequests.

The Chelsea Hospital for Women has received from the Mercers' Company a special grant of £105 in aid of the rebuilding of the hospital.

Mr. Edwin Parker, of St. Domingo Grove, Liverpool, bequeathed £100 to the Birkenhead Borough Hospital, and £100 each to the following institutions in Liverpool: The Stanley Hospital, Royal Southern Hospital, Convalescent Institution, General Infirmary, Home for Incurables, and Infirmary for Children.

Rates of Subscription: Twelve months, 6/6; Six months, 4/-; Three months, 2/-, post free to any address in the United Kingdom. Abroad, Twelve months, 8/8; Six months, 5/-; Three months, 2/6, post free.—Address The Subscription Dept., "The Hospital," The Hospital Building, 28/29 Southampton Street, Strand, London, W.C.

